# Commensurate incidence and outcomes of liver enzyme elevation between anti-tumor necrosis factor users with or without prior hepatitis B virus infections

**DOI:** 10.1371/journal.pone.0196210

**Published:** 2018-04-25

**Authors:** Ying-Ming Chiu, Mei-Shu Lai, K. Arnold Chan

**Affiliations:** 1 Division of Allergy, Immunology and Rheumatology, Changhua Christian Hospital, Changhua City, Taiwan; 2 Department of Nursing, College of Medicine and Nursing, Hungkuang University, Taichung City, Taiwan; 3 Graduate Institute of Epidemiology and Preventive Medicine, College of Public Health, National Taiwan University, Taipei, Taiwan; 4 Department of Medical Research, National Taiwan University Hospital, Taipei, Taiwan; 5 Graduate Institute of Oncology, College of Medicine, National Taiwan University, Taipei, Taiwan; Academia Sinica, TAIWAN

## Abstract

**Background and objective:**

Potential hepatoxicity is an important clinical concern when administering immunosuppressive therapies to patients infected by hepatitis B virus (HBV). Tumor necrosis factor inhibitors (anti-TNF) increase the likelihood of hepatitis consequent to HBV reactivation, but reported risks and outcomes vary. We determined the risks of liver enzyme elevation in anti-rheumatic drug users from an HBV-endemic region with differing HBV serostatus.

**Methods:**

We established retrospective cohorts with rheumatoid arthritis, ankylosing spondylitis, or psoriasis/psoriatic arthritis who: 1) received anti-TNF agents from 1 January 2004 to 30 June 2013; 2) received care from 1 June 2011 to 30 June 2013 but only ever used conventional disease-modifying anti-rheumatic drugs (DMARDs). Serology results defined three subgroups: HBV surface antigen positive (HBsAg+), HBsAg negative/HBV core antibody positive (HBsAg−/HBcAb+), or uninfected. We compared incidences of serum alanine aminotransferase (ALT) exceeding twice the upper reference limit between HBV serostatus subgroups in each treatment cohort.

**Results:**

Among 783 patients treated with anti-TNF (n = 472) or DMARDs only (n = 311), HBsAg−/HBcAb+ anti-TNF users had incidence of ALT elevation commensurate with uninfected counterparts (6.1 vs. 6.0/100 person-years), compared to 19.6/100 person-years in HBsAg+ patients (standardized rate ratio 3.3, 95% CI 1.3–8.2); none effected had severe or fatal hepatitis and ALT levels in all HBsAg−/HBcAb+ patients remained stable, mostly normalizing spontaneously, or after moderating treatment. Patterns of of ALT elevation associated with differing HBV serostatus in the DMARD cohort, resembled those in anti-TNF users.

**Conclusions:**

In this large HBV-endemic cohort, the absolute incidence of ALT elevation in anti-TNF users was more than three-fold higher in HBsAg+ patients than in uninfected counterparts; however, no such association was evident in patients with HBsAg−/HBcAb+ serotype, whose risk and outcomes of liver enzyme elevation were similar to uninfected patients, suggesting that anti-TNF use by HBsAg−/HBcAb+ patients is probably safe.

## Introduction

Use of immunosuppressant drugs, particularly methotrexate and leflunomide, in patients with immune-mediated diseases has been associated with hepatotoxicity, with severity ranging from asymptomatic to life-threatening or fatal [1, 2]. Such hepatotoxicity is especially frequent in patients with chronic liver diseases–notably hepatitis B virus (HBV) infection [3]. In individuals who have been infected by HBV, immunosuppressive agents can induce viral reactivation and serious clinical complications such as hepatitis, liver decompensation, or death, both in HBV surface antigen positive (HBsAg+) carriers and, more rarely, in those who are HBsAg-negative (HBsAg−) and HBV core antibody positive (HBcAb+) [4–6]. More than one-third of the world population may have been infected with HBV, three-quarters of whom live in countries of South-East Asia or the Western Pacific [7]. Taiwan is an HBV endemic country where 15–20% of the population are HBsAg+ and HBcAb seroprevalence was 80–90% before universal immunization of newborns against HBV began in 1986 [8–10]; therefore, the use of immunosuppressant therapies, particularly HBV-endemic regions such as Taiwan, warrants heightened clinical attention, including HBV screening, with antiviral prophylaxis for patients at high risk for HBV reactivation [11–15].

Tumor necrosis factor inhibitors (anti-TNF), including infliximab, etanercept, adalimumab, and golimumab, are biological agents that are indicated for treating various immune-mediated disorders. TNF inhibits HBV replication and stimulates HBV-specific T-cell responses that are important in clearing HBV from infected hepatocytes [16]. Inhibiting TNF suppresses the antiviral defense mechanism, thereby enabling increased viral replication [17]. However, the risk of HBV reactivation in HBsAg−/HBcAb+ patients receiving anti-TNF therapy is uncertain [12–14, 18–22]; there have been reports of HBV reactivation with fulminant hepatitis that necessitated antiviral therapy in such patients and even fatal hepatic failure [13, 18, 23–25], whereas others have found anti-TNF to be generally safe in this setting, even without anti-HBV prophylaxis [14, 19–22, 26]. In a systematic review of 257 HVB-infected patients treated with anti-TNF agents, HBV was reactivated in 5% with HBsAg−/HBcAb+ serostatus and 39% of HBsAg+ carriers [27]. Although the risks of immunosuppressive therapy in HBsAg+ patients are better known and hundreds of cases of HBV reactivation in HBsAg+ carriers treated with TNF inhibitors have been reported, estimated risks of hepatitis due to HBV reactivation vary widely, likely due to relatively small sample sizes, lack of prospective data, different immune-mediated diseases being evaluated between studies, and confounding effects of other immunosuppressants, such as corticosteroids [12, 14, 15, 23, 28–30].

Large scale studies of anti-TNF safety in patients with HBsAg+ or HBsAg−/HBcAb+ serostatus are rare [12–15, 22]; very few have analyzed data from hundreds of patients with common immune-mediated diseases who received anti-TNF therapies in real world clinical practice, and none has compared the risk of liver damage between HBsAg−/HBcAb+ and uninfected subjects. Hence, this study evaluated the overall incidence of liver enzyme elevation in anti-TNF-treated patients with various immune-mediated diseases and different HBV infection statuses, including both active carriers and those whose HBV serostatus indicated an infection at some time in the past, and to compare the absolute risks with those of patients without HBV infection.

## Patients and methods

### Study design and patient identification

**Fig 1:**
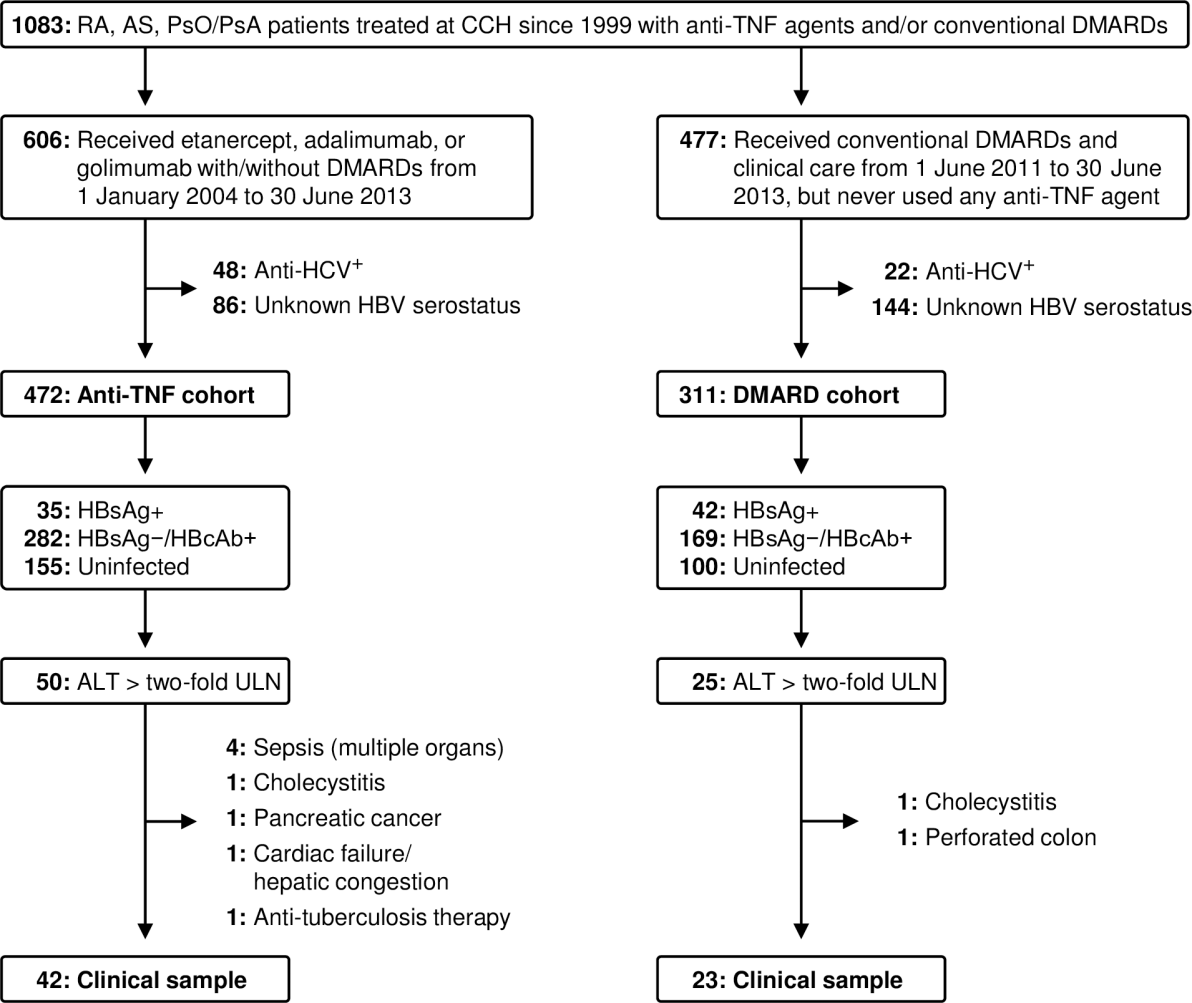
Selection and disposition of anti-TNF and DMARD cohorts. TNF, tumor necrosis factor; DMARD, disease-modifying anti-rheumatic drug; RA, rheumatoid arthritis; AS, ankylosing spondylitis; PsO/PsA, psoriasis/psoriatic arthritis; CCH, Changhua Christian Hospital; HCV, hepatitis C virus; HBV, hepatitis B virus; HBsAg+, HBV surface antigen positive; HBsAg−/HBcAb+, HBV surface antigen negative/HBV core antibody positive; ULN, upper limit of normal.

Changhua Christian Hospital is a 1,600-bed medical center in central Taiwan; from hospital records, we identified patients since 1999 with rheumatoid arthritis (RA), ankylosing spondylitis (AS), psoriasis or psoriatic arthritis (PsO/PsA), who all fulfilled international diagnostic criteria for these conditions (Fig 1). A retrospective anti-TNF-α cohort comprised an extant database of patients treated with etanercept, adalimumab, or golimumab from 1 January 2004 (when anti-TNF therapy for RA was first approved in Taiwan) through 30 June 2013. All patients starting anti-TNF therapy had previously received conventional DMARDs, including methotrexate, sulfasalazine, leflunomide, cyclosporine, hydroxychloroquine or cyclophosphamide, and most of them continued DMARDs concomitantly with anti-TNF. During this time, most patients with HBsAg+ serostatus had not received antiviral prophylaxis. A reference DMARD-only cohort was established from patients in the same pool who had only received conventional DMARDs from 1999 onward, with no anti-TNF therapy. To obtain the most recent clinical information, the DMARD-only cohort was restricted to eligible patients who received clinical care between 1 June 2011 and 30 June 2013. There was no overlap between patients in the anti-TNF and DMARD-only cohorts.

Anti-TNF cohort data were excerpted starting from the date of the first anti-TNF prescription, and DMARD-only cohort data from the first DMARD prescription after 1 January 1999. Follow-up of each treatment cohort discontinued upon the earliest occurrence of: death; development of liver enzyme elevation; discontinuation of the current regimen (defined as 3 months without refilling the medication prescription that qualified the individual for cohort entry); or 30 June 2013.

Changhua Christian Hospital Institution Review Board approved the study protocol (#101121); as it was a retrospective chart review and potentially identifying patient data were delinked to protect participants’ anonymity, the requirement for patient consent was waived. Study procedures were carried out in accordance with relevant guidelines and regulations.

### Hepatitis infection status

Patients’ HBV and hepatitis C virus (HCV) infection status were ascertained from medical records, based on the results of chemiluminescent microparticle immunoassays (Architect i2000sr System, Abbott Laboratories, Abbott Park, Illinois, USA). Patients whose serum had not been tested for HBV, or had tested positive for HCV antibody, were excluded. Based on HBV serology assay results, patients in each treatment cohort were divided into three HBV infection status categories: 1) HBcAb+ and HBsAg+, denoted HBsAg+; 2) HBcAb+ but HBsAg−, denoted HBsAg−/HBcAb+; or 3) both HBsAg− and HBcAb−, denoted uninfected.

### Outcome of interest–liver function

As a condition of receiving National Health Insurance reimbursed anti-TNF therapy, patients provided blood samples for liver function evaluation 6 months after commencing treatment and every 3 months thereafter. Serum alanine aminotransferase (ALT) elevations higher than one-fold, two-fold and three-fold the upper limit of normal (ULN) were analyzed as outcomes, with liver enzyme elevation operationally defined, as in another study of hepatotoxicity associated with anti-TNF therapy in RA [31], as serum ALT exceeding two-fold the upper reference limit of 40 international units/L.

#### Causes of liver enzyme elevation and clinical characteristics of the affected patients

Medical records of patients with serum ALT exceeding two-fold ULN were evaluated. After identifying patients whose existing medical conditions contributed to ALT elevation, we reviewed records of the remaining patients to ascertain their history of liver function tests, hepatic and medication profiles when liver function became abnormal, and the clinical course after developing ALT elevation.

### Data analysis and statistics

Incidence rates of ALT elevation were estimated by dividing the number of patients with ALT elevation by total person-years under treatment, having excluded cases potentially associated with an existing clinical condition. Age-gender-standardized incidences for each study group were calculated using the age and gender distributions of uninfected patients in the anti-TNF cohort as references. We calculated crude and adjusted rates, rate ratios, and 95% confidence intervals according to established epidemiological methods [32]. All analyses were performed using SAS® software, Version 9.2 for Windows (Copyright 2002–2008 SAS Institute Inc., Cary, NC, USA).

## Results

### Study subjects’ baseline characteristics and analysis of HBV infection status

From 1 January 2004 through 30 June 2013, 606 patients began treatment with anti-TNF agents at Changhua Christian Hospital, of whom 86 had insufficient serology data to define their HBV status and 48 tested anti-HCV positive (Fig 1). Most of the remaining 472 constituting the anti-TNF cohort (S1 Data Appendix) had also received prednisolone (83%) and concomitant DMARDs: methotrexate (72%); sulfasalazine (79%); hydroxychloroquine (65%); cyclosporine (33%); leflunomide (11%). Most started anti-TNF therapy before the Taiwan Rheumatology Association issued formal risk-management guidelines [11], and consequently only seven HBsAg+ patients had received antiviral agents either prior to, or concurrent with, their anti-TNF therapy; none of them had HBV reactivations during study follow-up (S1 Table), and only one (first row patient in S1 and S2 Tables) developed serum ALT levels exceeding twice ULN qualifying inclusion in S2 Table, which besides lists only those patients who received antiviral agents therapeutically, after developing liver enzyme elevation.

The non-biologic DMARD cohort comprised 311 patients (S1 Data Appendix) who had ongoing treatment from 1 June 2011 through 30 June 2013, excluding 144 with unknown HBV infection status and 22 with HCV antibodies. None had ever received anti-TNF agents.

Tables 1 and 2 summarize the characteristics of each treatment cohort according to HBV infection status. Females predominated in each treatment cohort and HBV infection category, and RA was the most common immune-mediated disease.

**Table 1:** Characteristics of the anti-TNF cohort.

		Patient numbers (proportions)			
HBV serostatus		HBsAg+	HBsAg−/HBcAb+	Uninfected	Totals
**Number** (proportion)		35 (7.4%)	282 (59.8%)	155 (32.8%)	**472 (100%)**
**Sex**					
Female		21 (60.0%)	192 (68.1%)	92 (59.4%)	**305 (64.6%)**
Male		14 (40.0%)	90 (31.9%)	63 (40.6%)	**167 (35.4%)**
**Age** (years)					
Median (range)		52.5 (24.1–78.9)	54.7 (25.8–85.6)	39.8 (6.6–78.8)	**52.1** **(6.6–85.6)**
**Disease**					
Rheumatoid arthritis		25 (71.4%)	217 (77.0%)	94 (60.6%)	**336 (71.2%)**
Ankylosing spondylitis		8 (22.9%)	39 (13.8%)	39 (25.2%)	**86 (18.2%)**
Psoriasis/Psoriatic arthritis		2 (5.7%)	26 (9.2%)	22 (14.2%)	**50 (10.6%)**
**Antiviral therapy**	Prophylactic^a^	3 (8.6%)	0	0	**7 (1.5%)**
	Preemptive^b^	4 (11.4%)	0	0	

TNF, tumor necrosis factor; HBV, hepatitis B virus; HBsAg+, HBV surface antigen positive; HBsAg−/HBcAb+, HBV surface antigen negative/HBV core antibody positive.

^a^ Prophylaxis = HBV DNA negative, liver function normal.

^b^ Preemptive = HBV DNA positive, liver function normal

**Table 2:** Characteristics of the DMARD cohort.

	Patient numbers (proportions)			
HBV serostatus	HBsAg+	HBsAg−/HBcAb+	Uninfected	Totals
**Number** (proportion)	42 (13.5%)	169 (54.3%)	100 (32.2%)	**311 (100%)**
**Sex**				
Female	26 (61.9%)	111 (65.7%)	62 (62.0%)	**199 (64.0%)**
Male	16 (38.1%)	58 (34.3%)	38 (38.0%)	**112 (36.0%)**
**Age** (years)				
Median (range)	44.6 (23.6–77.2)	52.3 (22.3–78.9)	44.5 (16.9–82.5)	**50.3** **(16.9–82.5)**
**Disease**				
Rheumatoid arthritis	23 (54.8%)	141 (83.4%)	64 (64.0%)	**228 (73.3%)**
Ankylosing spondylitis	15 (35.7%)	15 (8.9%)	26 (26.0%)	**56 (18.0%)**
Psoriasis/Psoriatic arthritis	4 (9.5%)	13 (7.7%)	10 (10.0%)	**27 (8.7%)**

DMARD, disease modifying anti-rheumatic drug; HBV, hepatitis B virus; HBsAg+, HBV surface antigen positive; HBsAg−/HBcAb+, HBV surface antigen negative/HBV core antibody positive.

### Incidence of liver enzyme elevation in patients with different HBV infection statuses

#### Anti-TNF cohort

The incidence of ALT elevation was significantly higher in HBsAg+ patients than among uninfected patients (Table 3), with at least three-fold difference in both crude and age-gender adjusted rate ratios. In contrast, rates of ALT elevation in HBsAg−/HBcAb+ patients were commensurate with those of uninfected patients.

**Table 3:** Incidence of liver enzyme elevation in anti-TNF cohort patients with different HBV serostatus.

HBV status (number)	Person- years	Cases of serum ALT >2-fold ULN (n = 42)^a^	Incidence of ALT elevation per 100 person-years, and rate ratios (95% confidence interval)			
			Crude		Standardized^b^	
			Incidence	Rate ratio	Incidence	Rate ratio
**All HBsAg+** (35)	47.1	9	19.1 (6.6–13.1)	3.2 (1.4–7.4)	19.6 (5.2–34.1)	3.3 (1.3–8.2)
**HBsAg+** excluding antiviral therapy^c^ (28)	35.6	8^d^	22.5 (6.9–38.0)	3.4 (1.4–8.2)	29.8 (5.3–34.3)	3.0 (1.2–7.5)
**HBsAg−/HBcAb+** (282)	451.3	20	4.4 (2.5–6.4)	0.7 (0.4–1.5)	6.1 (2.3–9.8)	1.0 (0.4–2.3)
**Uninfected** (155)	216.0	13	6.0 (2.7–9.3)	Reference	6.0 (2.7–9.3)	Reference

TNF, tumor necrosis factor; HBV, hepatitis B virus; ALT, alanine aminotransferase; ULN, upper limit of normal; HBsAg+, HBV surface antigen positive; HBsAg−/HBcAb+, HBV surface antigen negative/HBV core antibody positive.

^a^ Excluding eight cases attributed to existing clinical conditions.

^b^ Adjusted incidence in HBsAg+ and HBsAg−/HBcAb+ patients standardized by age and gender using uninfected patients in the anti-TNF-α cohort as the reference.

^c^ Prophylactic or preemptive prior to developing ALT elevation.

^d^ Cases of serum ALT >2-fold ULN becomes n = 41, excluding one anti-TNF user (first row of S1 & S2 Tables) who developed ALT elevation after starting antiviral therapy.

#### DMARD cohort

The incidence of ALT elevation was significantly higher among HBsAg+ patients than in uninfected patients (Table 4), whereas there was no statistically significant difference between the incidence rates of those with HBsAg−/HBcAb+ serostatus and uninfected patients.

**Table 4:** Incidence of liver enzyme elevation in DMARD cohort patients with different HBV serostatus.

HBV status (number)	Person- years	Cases of serum ALT >2-fold ULN (n = 23)^a^	Incidence of ALT elevation per 100 person-years, and rate ratios (95% confidence interval)			
			Crude		Standardized^b^	
			Incidence	Rate ratio	Incidence	Rate ratio
**HBsAg+** (42)	72.0	11	15.3 (6.2–24.3)	5.7 (1.8–18.0)	22.6 (4.2–41.0)	10.3 (2.7–39.8)
**HBsAg−/HBcAb+** (169)	373.5	8	2.1 (0.7–3.6)	0.8 (0.2–2.7)	8.1 (0.7–15.6)	3.7 (1.0–14.3)
**Uninfected** (100)	149.8	4	2.7 (0.1–5.3)	Reference	2.2 (−0.2–4.6)	Reference

DMARD, disease modifying anti-rheumatic drug; HBV, hepatitis B virus; ALT, alanine aminotransferase; ULN, upper limit of normal; HBsAg+, HBV surface antigen positive; HBsAg−/HBcAb+, HBV surface antigen negative/HBV core antibody positive.

^a^ Excluding two cases attributed to existing clinical conditions.

^b^ Adjusted incidence in HBsAg+ and HBsAg−/HBcAb+ patients standardized by age and gender using uninfected patients in the anti-TNF-α cohort as the reference.

### Causes of liver enzyme elevation and clinical characteristics of the affected patients

#### Anti-TNF cohort

Eight of 50 patients with serum ALT exceeding two-fold ULN had a clinical condition that could explain liver enzyme elevation: cholecystitis (n = 1), pancreatic cancer (n = 1), sepsis involving multiple organs (n = 4), cardiac failure with hepatic congestion (n = 1), and a patient receiving treatment for tuberculosis (n = 1). Among the remaining 42 (Table 5; S2 Table), 79% had their first occurrence of abnormal liver function during the first year of anti-TNF treatment. Ten had history of liver function impairment. Most (39/42) were receiving concurrent DMARDs when liver function became abnormal: specifically, 28 (66.7%) received methotrexate at a mean dose of 10.2 mg/week (range 2.5–15.0 mg/week), either with or without other DMARDs, of whom 11 did not take folic acid, which can ameliorate methotrexate hepatotoxicity. Besides methotrexate, two were receiving leflunomide when ALT elevation developed, and another azathioprine. Apart from hepatotoxicity caused by immunosuppressant drugs, other causes of liver enzyme elevation among these 42 patients included: hepatitis consequent to HBV reactivation; transient and spontaneous ALT elevation; and chronic liver disease (operationally defined as persistent ALT elevation without, or after, discontinuing hepatotoxic drugs and with no evidence of HBV reactivation). None had fulminant hepatitis or died due to hepatic failure after ALT elevation was ascertained.

**Table 5:** Clinical status of patients with abnormal liver function during anti-TNF therapy.

Patient disposition (n = 42)		Number of patients (proportion)
**Age** at which liver enzyme elevation detected (mean)		45.3 years
**HBV status**		
HBsAg+		9 (21.4%)
HBsAg−/HBcAb+		20 (47.6%)
Uninfected		13 (31.0%)
**Prior abnormal liver function**		10 (23.8%)
**Anti-TNF agent** when liver enzyme elevation arose		42 (100.0%)
Etanercept		28 (66.7%)
Adalimumab		13 (31.0%)
Golimumab		1 (2.4%)
**DMARD(s)** when liver enzyme elevation arose		39 (92.9%)
Methotrexate	with folic acid	17 (40.5%)
	without folic acid	11 (26.2)
Sulfasalazine		24 (57.1%)
Hydroxychloroquine		15 (35.7%)
Cyclosporine		3 (7.1%)
Leflunomide		2 (4.8%)
Azathioprine		1 (2.4%)
**Outcome**		
serum ALT normalized:	after moderating drug regimen	26 (61.9%)
	spontaneously	12 (28.6%)
Serum ALT abnormality persisted		4 (9.5%)

TNF, tumor necrosis factor; DMARD, disease-modifying anti-rheumatic drug; HBV, hepatitis B virus; HBsAg+, HBV surface antigen positive; HBsAg−/HBcAb+, HBV surface antigen negative/HBV core antibody positive; ALT, alanine aminotransferase.

ALT levels normalized in one HBsAg+ patient already taking long-term antiviral therapy whose medications were not adjusted, and in three others who were changed to a less intensive drug regimen and started on standard antiviral therapies in response to ALT elevation (S2 Table). In most other cases, irrespective of HBV status, serum ALT returned to normal either spontaneously, without altering the drug regimen (11/42), or after withdrawing anti-TNF agents and/or moderating the conventional DMARD regimen (23/42). However, serum ALT did remain persistently abnormal in two uninfected patients and one HBsAg−/HBcAb+ patient whose regimens were not adjusted, and in another uninfected patient who stopped receiving methotrexate but stayed on anti-TNF therapy (adalimumab). No HBsAg−/HBcAb+ or HBsAg+ patients with liver enzyme elevation had adverse hepatic sequelae.

#### DMARD cohort

Causes of liver enzyme elevation were similar to those in the anti-TNF cohort (S3 Table); however, none had a history of ALT elevation. Management and outcomes were also similar to those of the anti-TNF cohort.

## Discussion

We investigated and compared the incidence of liver enzyme elevation among 783 patients from Taiwan with common immune-mediated diseases and different HBV infection statuses, who were treated with either anti-TNF agents, or only non-biologic DMARDs. Among anti-TNF users, HBsAg−/HBcAb+ patients had incidence and clinical outcomes of ALT elevation commensurate with those of uninfected patients, whereas HBsAg+ patients had more than three-fold higher relative risk.

To our knowledge, this is the largest clinical epidemiology study of hepatic outcomes according to HBV serostatus among patients who received immunosuppressive anti-rheumatic therapies in real-world clinical practice. Conducting this study in an HBV-endemic country enabled us to identify large cohorts of patients with different HBV infection statuses, including both HBsAg−/HBcAb+ and HBsAg+/HBcAb+. And unlike previous studies that included only one or two immune-mediated diseases [14, 15, 22, 23, 28–30] we included patients with several common immune-mediated diseases treated with anti-TNF agents; consequently, the results may be more representative of the real-world risk of ALT elevation confronting rheumatologists in clinical practice. Besides, we have accrued numerous clinical cases that provide useful information for clinicians (S1 Data Appendix, S2 and S3 Tables).

### Incidence of liver enzyme elevation

#### Anti-TNF cohort

Previous studies have reported conflicting results concerning the risk of HBV reactivation and hepatitis in HBsAg−/HBcAb+ patients [13, 18–26]. In this large endemic cohort, the incidence of ALT elevation in HBsAg−/HBcAb+ patients was 6.1/100 person years, similar to that in uninfected patients, with comparable clinical outcomes and no severe hepatic sequelae. The absence of fulminant hepatitis or fatal hepatic failure in this anti-TNF cohort contrasts with other studies [13, 18, 23–27]; among 25 published reports of HBsAg+ rheumatic disease patients who received anti-TNF therapy without antiviral prophylaxis, 13 (52%) had HBV reactivations, leading to three cases of fulminant hepatitis, a liver transplant, and one death [26]. Antiviral therapy preceding, or concurrent with, anti-TNF in some patients may also explain why the incidence of HBV reactivation among HBsAg+ patients appeared lower than other studies in which antiviral prophylaxis was not used. Higher rates of hepatic complications among HBsAg−/HBcAb+ patients in other reports might be due to intensive treatment with immunomodulatory agents or corticosteroids that had increased the viral load before anti-TNF therapy commenced [23].

HBV reactivation in HBsAg−/HBcAb+ patients is usually defined as the reappearance of HBsAg or an increase in the level of HBV DNA while liver function is still normal [18]; however, HBV DNA levels may fluctuate between detectable and undetectable, without any other clinical manifestations [22]. In the absence of viral load data, we may have missed cases of HBV reactivation in HBsAg−/HBcAb+ patients with ALT elevation. Estimated incidence of HBV reactivation in HBsAg−/HBcAb+ patients receiving anti-TNF-α therapies ranges from ~1–5% [12, 22, 27], and was 1.7% in a meta-analysis of 486 such patients [33]; given this low incidence, our sample size of 282 may also have been too small to detect such an infrequent occurrence. Nonetheless, all such patients in our chart review made good recoveries despite receiving no antiviral therapy, which suggests that even if some cases of HBV reactivation without ALT elevation did occur, these were probably mild and had no adverse hepatic consequences, as was observed in some other recent studies [14, 22]. Our findings support the proposition that anti-TNF agents can generally be prescribed safely to HBsAg−/HBcAb+ patients [19–22, 26].

Three-fold higher age-gender-adjusted incidence of ALT elevation among HBsAg+ patients treated with anti-TNF agents than in their uninfected counterparts may relate to underlying chronic liver disease, HBV reactivation related to immunosuppression, or to both. First, since HBsAg+ patients are more likely to develop chronic liver disease and cirrhosis, they are more likely to manifest ALT elevation. Second, HBsAg+ patients have a higher risk than uninfected patients of liver enzyme elevation when exposed to hepatotoxic drugs such as methotrexate (synergistic effect) [3]. Moreover, the use of immunosuppressive agents in HBsAg+ patients may induce acute hepatitis due to HBV reactivation that manifests in elevated ALT levels [13, 15, 27]. There may also be a high risk of liver enzyme elevation during the first few months after discontinuing anti-TNF or other immunosuppressive therapies, due to immunologic flare as immune competence is restored and infected hepatocytes are rapidly destroyed [12, 34, 35] Therefore, we followed-up the liver function of HBsAg+ patients in both cohorts for 12 months after treatment discontinued; however, we observed no evidence of acute hepatitis flares.

#### DMARD cohort

We did not directly compare the two treatment cohorts, because the conventional DMARD-only cohort was artificial. As a corollary of not being prescribed anti-TNF therapy, the disease state of this group of patients must have been deemed less severe, and was therefore unlikely to be treated with potent immunosuppressive agents, such as methotrexate, that are known to be hepatotoxic. Furthermore, year of cohort entry, age, sex, and immune-mediated disease, differed between patients in each cohort with the same HBV infection status. Nevertheless, in both treatment cohorts HBsAg+ patients had significantly higher incidence of ALT elevation than HBsAg−/HBcAb+ or uninfected patients. These observations suggest that hepatotoxic medications and the type of immune-mediated disorder are both important factors associated with liver enzyme elevation.

### Study limitations

Our study had noteworthy limitations. Foremost, hepato-virologic data or liver biopsy findings were mostly lacking, which prevented us from distinguishing between liver damage arising from HBV reactivation as opposed to confounding factors such as alcohol or drug toxicity, or fatty-liver disease, or to compare HBV reactivation rates between patient subgroups. Since we had no non-treated control group, we do not know the comparative risk of liver enzyme elevation in HBV-infected patients not receiving immunotherapies. Second, it is probable that different doses and types of immunosuppressive agents in different HBV infection status categories affected the incidence of liver enzyme elevation. Drug-induced hepatotoxicity may have also differed between subgroups treated with different regimens. Awareness about the risk of HBV reactivation associated with anti-TNF may cause physicians in Taiwan to prescribe anti-TNF agents to only those HBsAg+ patients that they believe to be at lower risk of HBV reactivation. They may also avoid prescribing hepatotoxic drugs to HBsAg+ patients, or give medications at lower doses. Such circumstances may lead the risk of liver enzyme elevation in HBsAg+ patients to be underestimated.

We followed the majority of previous reports in applying the single criterion of HBsAg−/HBcAb+ serostatus to define past infection. Some studies have shown higher risk for reactivation in patients with HBsAg−/HBcAb+ and HBV surface antibody positive serotype; however, we had no HBV surface antibody data to perform such subgroup analysis. Lastly, use of antiviral drugs either before or during anti-TNF therapy would also be expected to lead the incidence of liver enzyme elevation to be underestimated; however, only seven HBsAg+ patients in our study received anti-hepatitis drugs because formal risk management guidelines were not issued in Taiwan until 2012 [11].

### Conclusions

This large cohort study in an HBV-endemic region reflects the ‘real-world’ risk of hepatotoxicity in HBV-infected patients receiving immunotherapy for common rheumatic diseases. Compared with uninfected patients, HBsAg−/HBcAb+ patients treated with anti-TNF agents had similar risk of developing ALT elevation, whereas the risk among HBsAg+ patients was at least three-fold higher. Causes of ALT elevation included hepatitis consequent to HBV-reactivation, chronic liver disease, and drug-induced hepatotoxicity. No cases of liver enzyme elevation in HBsAg−/HBcAb+ anti-TNF users without antiviral therapy led to severe hepatitis or acute liver failure, and serum ALT levels in most such patients returned to normal either spontaneously, or after moderating the DMARD regimen and/or withdrawing anti-TNF agents; therefore, anti-TNF use by HBsAg−/HBcAb+ patients is probably safe.

## Supporting information

S1 Data Appendix Anti-TNF and DMARD cohorts: Disease, HBV, and ALT status and clinical data. (XLSX)

S1 Table Clinical characteristics and outcomes of patients who received antiviral therapy. (PDF)

S2 Table Clinical status of anti-TNF users who developed ALT elevation. (PDF)

S3 Table Clinical status of DMARD users who developed ALT elevation. (PDF)
