# Supplementary material for: Commensurate incidence and outcomes of liver enzyme elevation between anti-tumor necrosis factor users with or without prior hepatitis B virus infections
Source: PLoS One. 2018 Apr 25;13(4):e0196210. doi: 10.1371/journal.pone.0196210 (PMC5919014; doi:10.1371/journal.pone.0196210)
Supplement: S1 Table — (PDF) [file pone.0196210.s002.pdf]

Table S1. Clinical characteristics of anti-TNF cohort patients who received antiviral therapy[a], and its outcomes

Disease	HBV load (IU/ml)	Liver function	Treatment (start date)	Antiviral therapy		Outcome
				Regimen	Duration	
Rheumatoid arthritis	Negative	Normal	Golimumab (27/7/2012)	Lamivudine 100 mg (prophylaxis)<br>Telbivudine 600 mg (prophylaxis)	11/3/2011–9/9/2015<br>9/9/2015–	No HBV reactivation since starting antiviral drug
Ankylosing spondylitis	Negative	Normal	Adalimumab (28/1/2013)	Entecavir 0.5 mg (prophylaxis[b])	28/1/2013–	No HBV reactivation since starting antiviral drug
Psoriatic arthritis	24,664	Normal	Adalimumab (17/5/2013)	Lamivudine 100 mg (preemptive[c])	3/5/2013–5/9/2014	HBV load 540 IU/ml at 27/9/2014<br>Anti-TNF withdrawn 21/11/2014
Ankylosing spondylitis	361,893,284	Normal	Adalimumab (4/3/2010)	Lamivudine 100 mg (preemptive)	18/3/2010–22/4/2010	Discontinued treatments 22/4/2010<br>Normal liver function after stopping lamivudine
Rheumatoid arthritis	181,915	Normal	Etanercept (12/4/2007)	Entecavir 0.5 mg (preemptive)[d]	13/5/2013–	Undetectable HBV load since starting antiviral drug
Rheumatoid arthritis	13,519	Normal	Adalimumab (21/1/2011)	Entecavir 0.5 mg (preemptive)[d]	4/1/2014–	Undetectable HBV load since starting antiviral
Rheumatoid arthritis	8,794	Normal	Adalimumab (26/3/2011)	Entecavir 0.5 mg (preemptive)[d]	5/2/2013–	Undetectable HBV load since starting antiviral drug

TNF, tumor necrosis factor; HBV, hepatitis B virus; IU, international units.

[a] Until study follow-up discontinued.

[b] Prophylaxis = HBV DNA negative, liver function normal.

[c] Preemptive = HBV DNA positive, liver function normal.

[d] Started preemptive antiviral therapy following issuance of Taiwan Rheumatology Association guidelines.

Ying-Ming Chiu, Mei-Shu Lai, K. Arnold Chan. Commensurate incidence and outcomes of liver enzyme elevation between anti-tumor necrosis factor users with or without prior hepatitis B virus infections. PLOS ONE 2018: DOI: 10.1371/journal.pone.0196210
