# Supplementary material for: Commensurate incidence and outcomes of liver enzyme elevation between anti-tumor necrosis factor users with or without prior hepatitis B virus infections
Source: PLoS One. 2018 Apr 25;13(4):e0196210. doi: 10.1371/journal.pone.0196210 (PMC5919014; doi:10.1371/journal.pone.0196210)
Supplement: S2 Table — (PDF) [file pone.0196210.s003.pdf]

Table S2. Clinical status of 42 patients who developed liver enzyme elevation during treatment with anti-TNF agents[a]

Disease	HBV serostatus		Prior ALT elevation[b]	Hepatic profile when liver enzyme elevation arose					Medication profile when liver enzyme elevation arose						Management response to ALT elevation	Serum ALT after management response
	HBsAg	HBcAb		AST/ALT (x ULN)	Total bilirubin (mg/dl)	Coagulation	Virology	Liver sonography[c]	Anti-TNF agent	Anti-TNF duration (mo)	MTX dose (mg/wk)	MTX with folate	PRED dose (mg/dy)	Other DMARD(s)		
RA	+	+	No	NR/2.6	NR	NR	ND	Liver cirrhosis	GLM	5.6	0	NR	5	HCQ/SSZ	None. Keep concurrent antiviral therapy	Normal
AS	+	+	No	3.5/5.5	0.47	NR	HBV DNA: 6491 IU/ml HBeAg−, HBeAb+	Normal	ADA	3.7	2.5	Yes	0	SSZ	Stop ADA/MTX	Normal
RA	+	+	Yes	1.7/2.3	NR	NR	ND	PLD	ETA	0.7	7.5	Yes	7.5	HCQ/SSZ	Stop MTX, start antiviral therapy	Normal
RA	+	+	No	NR/2.0	NR	NR	ND	Normal	ETA	1.6	7.5	No	0	None	Stop ETA	Normal
RA	+	+	No	NR/2.4	NR	NR	ND	ND	ETA	22.4	5	Yes	5	HCQ/SSZ	None	Normal
RA	+	+	No	NR/2.3	NR	NR	ND	ND	ETA	0.9	7.5	No	5	SSZ	None	Normal
RA	+	+	No	2.2/3.7	NR	NR	HBeAg−	Normal	ETA	11.2	0	NR	5	LEF/HCQ/SSZ	Stop ETA/PRED/LEF	Normal
RA	+	+	No	22.6/33.9	1.68	PT: 11.4 INR: 1.2	HBV DNA: 186940 IU/ml HBeAg+	Fatty liver	ETA	4.7	10	Yes	2.5	HCQ/SSZ	Stop ETA/MTX, start antiviral therapy	Normal
RA	+	+	Yes	6.8/8.5	8.3	PT: 14.3 INR: 1.3	HBV DNA: 5522132 IU/ml HBeAg−	Normal	ETA	0.9	15	Yes	10	HCQ/SSZ	Stop MTX/ETA, start antiviral therapy	Normal
AS	−	+	No	NR/2.8	NR	NR	ND	Fatty liver	ADA	1.9	15	Yes	5	SSZ	Stop MTX	Normal
AS	−	+	No	NR/2.4	NR	NR	ND	ND	ADA	10.3	0	NR	0	None	None	Normal
RA	−	+	No	NR/2.5	NR	NR	ND	ND	ADA	12.0	15	Yes	5	HCQ	None	Normal
PsO	−	+	No	2.6/2.8	NR	NR	ND	PLD	ADA	1.9	10	No	0	SSZ	MTX 10→7.5 mg	Normal
AS	−	+	No	1.4/3.2	NR	NR	ND	Fatty liver	ADA	13.1	0	NR	0	SSZ	None	Abnormality persisted
RA	−	+	No	NR/3.2	NR	NR	HBV DNA: negative	PLD	ADA	7.5	15	No	7.5	None	MTX 15→10 mg	Normal
PsO	−	+	Yes	3.1/3.0	NR	NR	ND	PLD	ADA	7.5	0	NR	0	CYS	None	Normal
RA	−	+	Yes	NR/2.1	NR	NR	ND	Fatty liver	ETA	1.6	7.5	No	10	SSZ	Stop SSZ, add folate	Normal

Ying-Ming Chiu, Mei-Shu Lai, K. Arnold Chan. Commensurate incidence and outcomes of liver enzyme elevation between anti-tumor necrosis factor users with or without prior hepatitis B virus infections. PLOS ONE 2018. DOI: 10.1371/journal.pone.0196210

RA	−	+	Yes	NR/9.0	NR	NR	ND	Normal	ETA	6.3	10	No	5	HCQ/SSZ	Stop MTX	Normal
RA	−	+	No	NR/5.5	NR	NR	ND	PLD	ETA	1.9	10	No	7.5	HCQ/SSZ	MTX 10→5 mg	Normal
RA	−	+	Yes	NR/2.6	NR	NR	HBV DNA: negative	PLD	ETA	27.1	15	Yes	0	None	MTX 15→7.5 mg	Normal
RA	−	+	No	NR/2.2	NR	NR	ND	Fatty liver	ETA	39.7	15	Yes	2.5	None	None	Normal
RA	−	+	Yes	NR/2.4	NR	NR	ND	Normal	ETA	21.1	7.5	No	0	HCQ	MTX 7.5→5 mg	Normal
RA	−	+	No	NR/2.5	NR	NR	ND	ND	ETA	25.1	5	Yes	0	None	MTX 5→2.5 mg	Normal
RA	−	+	No	NR/2.2	NR	NR	ND	ND	ETA	8.4	10	Yes	5	SSZ	Stop ETA, MTX 10→7.5 mg	Normal
RA	−	+	No	NR/3.6	NR	NR	ND	Fatty liver	ETA	10.3	10	Yes	5	HCQ	MTX 10→5 mg	Normal
RA	−	+	Yes	1.6/2.1	NR	NR	ND	ND	ETA	6.5	10	Yes	0	HCQ/SSZ	MTX 10→5 mg	Normal
AS	−	+	No	NR/2.7	NR	NR	ND	ND	ETA	12.6	0	NR	0	SSZ	None	Normal
PsA	−	+	Yes	2.9/5.7	NR	NR	HBV DNA: negative	Normal	ETA	0.7	0	NR	5	HCQ/SSZ/CYS	Stop ETA	Normal
AS	−	+	No	NR/5.5	NR	NR	ND	Fatty liver	ETA	4.4	0	NR	0	SSZ	Stop ETA	Normal
PsA	−	−	No	NR/3.0	NR	NR	NR	ND	ADA	11.7	0	NR	5	CYS/LEF	None	Normal
AS	−	−	No	NR/3.7	NR	NR	NR	Fatty liver	ADA	21.0	10	Yes	0	None	Stop MTX	Abnormality persisted
RA	−	−	No	1.3/2.5	NR	NR	NR	Fatty liver	ADA	2.8	10	No	5	HCQ, SSZ	MTX 10→5 mg	Normal
RA	−	−	No	NR/2.3	NR	NR	NR	Fatty liver	ADA	15.9	10	Yes	0	SSZ	None	Normal
RA	−	−	No	NR/2.6	NR	NR	NR	ND	ADA	4.7	15	No	5	None	MTX 15→7.5 mg	Normal
RA	−	−	No	NR2.5	NR	NR	NR	ND	ETA	4.7	15	Yes	5	None	MTX 15→12.5 mg	Normal
JRA	−	−	No	NR/3.6	NR	NR	NR	Normal	ETA	7.6	10	No	5	AZA/HCQ	None	Normal
JRA	−	−	No	NR/2.2	NR	NR	NR	ND	ETA	3.7	5	Yes	1.25	None	None	Normal
AS	−	−	No	NR/2.0	NR	NR	NR	ND	ETA	4.7	0	NR	0	SSZ	SSZ 1 g→500 mg	Normal
AS	−	−	No	0.9/3.7	0.25	NR	NR	Normal	ETA	0.7	0	NR	0	SSZ	Stop ETA	Normal
AS	−	−	No	0.9/2.5	0.5	NR	NR	Fatty liver	ETA	14.7	0	NR	0	None	None	Abnormality persisted
AS	−	−	No	NR/7.1	NR	NR	NR	ND	ETA	4.4	0	NR	0	SSZ	SSZ 2 g→1 g	Normal

Ying-Ming Chiu, Mei-Shu Lai, K. Arnold Chan. Commensurate incidence and outcomes of liver enzyme elevation between anti-tumor necrosis factor users with or without prior hepatitis B virus infections. PLOS ONE 2018. DOI: 10.1371/journal.pone.0196210

PsO	–	–	Yes	1.5/3.2	NR	NR	NR	ND	ETA	11.2	0	NR	0	None	None	Abnormality persisted

TNF, tumor necrosis factor; mo, month; wk, week; dy, day; HBV, hepatitis B virus; HBsAg, HBV surface antigen; HBcAb, HBV core antibody; HBeAg, HBV e antigen; HBeAb, HBV e antibody; ALT/AST, alanine/aspartate aminotransferase; ULN, upper limit of normal; DMARDs, disease-modifying anti-rheumatic drugs; MTX, methotrexate; PRED, prednisolone; ETA, etanercept; ADA, adalimumab; GLM, golimumab; HCQ, hydroxychloroquine; SSZ, sulfasalazine; CYS, cyclosporine; AZA, azathioprine; LEF, leflunomide; RA, rheumatoid arthritis; AS, ankylosing spondylitis; PsO, psoriatic arthritis; JRA, juvenile rheumatoid arthritis; PLD, parenchymal liver disease[c]; PT, prothrombin time; INR, international normalized ratio; IU, international units; ND, not done; NR, not reported.

[a] Chart review included 42/50 patients treated with anti-TNF agents who had ALT >two-fold ULN; eight patients were excluded because their elevated ALT was due to other internal medicine or surgical problems.

[b] ALT >two-fold ULN during the past year of anti-TNF treatment.

[c] In Taiwan, ultrasound findings intermediate between "normal" and "cirrhosis" based on sonographic evaluation criteria for liver surface, liver parenchyma, hepatic vessels and spleen size, are diagnosed as "parenchymal liver disease". These criteria are described in detail in: Hung CH, Lu SN, Wang JH, et al. Correlation between ultrasonographic and pathologic diagnoses of hepatitis B and C virus-related cirrhosis. J Gastroenterol. 2003;38:153–7.

Ying-Ming Chiu, Mei-Shu Lai, K. Arnold Chan. Commensurate incidence and outcomes of liver enzyme elevation between anti-tumor necrosis factor users with or without prior hepatitis B virus infections. PLOS ONE 2018. DOI: 10.1371/journal.pone.0196210
