# Supplementary material for: Commensurate incidence and outcomes of liver enzyme elevation between anti-tumor necrosis factor users with or without prior hepatitis B virus infections
Source: PLoS One. 2018 Apr 25;13(4):e0196210. doi: 10.1371/journal.pone.0196210 (PMC5919014; doi:10.1371/journal.pone.0196210)
Supplement: S3 Table — (PDF) [file pone.0196210.s004.pdf]

Table S3. Clinical status of 23 patients who developed liver enzyme elevation during treatment with DMARDs[a]

Disease	HBV serostatus		Prior ALT elevation[b]	Hepatic profile when liver enzyme elevation arose					Medication profile when liver enzyme elevation arose					Management response to ALT elevation	Serum ALT after management response
				AST/ALT (x ULN)	Total bilirubin (mg/dl)	Coagulation	Virology	Liver sonography[c]	DMARDs		MTX		PRED dose (mg/dy)		
	HBsAg	HBcAb							drug(s) used	duration (mo)	dose (mg/wk)	with folate			
RA	+	+	No	5.8/6.2	1.93	PT: 12.2<br>INR: 1.16	HBV DNA: 16417047 IU/ml	PLD	HCQ/SSZ/CYS	0.0	0	NR	10	Stop PRED, start antiviral therapy	Normal
RA	+	+	No	NR/4.6	NR	NR	ND	Normal	LEF/HCQ/SSZ	0.0	0	NR	5	Stop LEF	Normal
RA	+	+	No	NR/4.2	NR	NR	ND	Normal	MTX/HCQ/SSZ	0.0	7.5	Yes	5	None	Normal
RA	+	+	No	NR/8.8	NR	NR	ND	Normal	MTX/HCQ/SSZ	0.0	7.5	Yes	5	Stop MTX	Normal
RA	+	+	No	15.0/32.0	1.57	NR	HBV DNA: 170 IU/ml<br>HBeAg−	Normal	MTX/HCQ/SSZ	0.0	7.5	No	5	Stop DMARDs, start antiviral therapy	Normal
RA	+	+	No	NR/2.0	NR	NR	ND	Fatty liver	MTX/HCQ/SSZ	0.0	10	Yes	5	None	Normal
RA	+	+	No	NR/2.6	NR	NR	HBeAg−	PLD	MTX/HCQ	0.0	7.5	Yes	2.5	MTX→5 mg, start antiviral therapy	Normal
RA	+	+	No	NR/6.1	NR	NR	ND	Normal	MTX/HCQ/SSZ	0.0	12.5	Yes	5	MTX→7.5 mg	Normal
AS	+	+	No	NR/2.2	NR	NR	ND	Fatty liver	SSZ	0.0	0	NR	0	None	Normal
AS	+	+	No	NR/2.2	NR	NR	HBeAg−, HBeAb+	Normal	HCQ/SSZ	0.0	0	NR	0	None	Normal
RA	+	+	No	NR/4.0	NR	NR	ND	ND	MTX/HCQ/SSZ	0.0	15	Yes	5	None	Normal
RA	−	+	No	1.4/2.7	NR	NR	ND	PLD	HCQ/SSZ	0.0	0	NR	10	None	Normal
AS	−	+	No	3.4/8.3	6.78	NR	ND	Fatty liver	SSZ	0.0	0	NR	0	Stop SSZ	Normal
AS	−	+	No	NR/2.2	0.98	NR	ND	ND	SSZ	0.0	0	NR	10	None	Normal
AS	−	+	No	NR/2.0	NR	NR	ND	Fatty liver	SSZ	0.0	0	NR	0.5	None	Abnormality persisted
RA	−	+	No	7.8/9.3	12.64	PT: 10.6<br>INR: 1.11	ND	PLD	MTX/AZA	0.0	7.5	Yes	5	Stop DMARDs	Normal
RA	−	+	No	NR/2.2	NR	NR	ND	ND	MTX/HCQ/SSZ	0.0	7.5	Yes	10	None	Normal
RA	−	+	No	NR/2.2	NR	NR	ND	ND	MTX/HCQ/SSZ/CYS	0.0	15	Yes	5	MTX→7.5 mg	Normal
RA	−	+	No	NR/2.8	NR	NR	ND	Normal	MTX/HCQ/SSZ/CYS	0.0	15	Yes	7.5	Stop MTX	Normal
AS	−	−	No	NR/2.4	NR	NR	NR	ND	SSZ	0.0	0	NR	0	None	Normal
RA	−	−	No	NR/2.6	NR	NR	NR	ND	MTX/HCQ/SSZ	0.0	10	No	2.5	Stop MTX	Normal
RA	−	−	No	NR/4.1	NR	NR	NR	PLD	MTX/HCQ/SSZ	0.0	15	No	5	MTX→7.5 mg	Normal
RA	−	−	No	NR/9.3	NR	NR	NR	NA	MTX/HCQ/SSZ/CYS	0.0	15	Yes	10	Stop MTX	Normal

Ying-Ming Chiu, Mei-Shu Lai, K. Arnold Chan. Commensurate incidence and outcomes of liver enzyme elevation between anti-tumor necrosis factor users with or without prior hepatitis B virus infections. PLOS ONE 2018. DOI: 10.1371/journal.pone.0196210

DMARDs, disease-modifying anti-rheumatic drugs; mo, month; wk, week; dy, day; HBV, hepatitis B virus; HBsAg, HBV surface antigen; HBcAb, HBV core antibody; HBeAg, HBV e antigen; HBeAb, HBV e antibody; ALT/AST, alanine/aspartate aminotransferase; ULN, upper limit of normal; MTX, methotrexate; PRED, prednisolone; LEF, leflunomide; HCQ, hydroxychloroquine; SSZ, sulfasalazine; CYS, cyclosporine; AZA, azathioprine; RA, rheumatoid arthritis; AS, ankylosing spondylitis; PLD, parenchymal liver disease[c]; PT, prothrombin time; INR, international normalized ratio; IU, international units; ND, not done; NR, not reported; NA, not applicable.

[a] Chart review included 23/25 DMARD-treated patients who had ALT >two-fold ULN; two patients were excluded because their elevated ALT was due to other internal medicine or surgical problems.

[b] ALT >two-fold ULN during the past year of DMARD treatment.

[c] In Taiwan, ultrasound findings intermediate between "normal" and "cirrhosis" based on sonographic evaluation criteria for liver surface, liver parenchyma, hepatic vessels and spleen size, are diagnosed as "parenchymal liver disease". These criteria are described in detail in: Hung CH, Lu SN, Wang JH, et al. Correlation between ultrasonographic and pathologic diagnoses of hepatitis B and C virus-related cirrhosis. J Gastroenterol. 2003;38:153–7.

Ying-Ming Chiu, Mei-Shu Lai, K. Arnold Chan. Commensurate incidence and outcomes of liver enzyme elevation between anti-tumor necrosis factor users with or without prior hepatitis B virus infections. PLOS ONE 2018. DOI: 10.1371/journal.pone.0196210
